# A case of granulomatous slack skin cutaneous T-cell lymphoma: PET/CT imaging findings

**DOI:** 10.1259/bjrcr.20150052

**Published:** 2015-05-05

**Authors:** A T Kendi, S Parker, D Parker, B Barron

**Affiliations:** ^1^Radiology and Imaging Sciences, Emory University, Atlanta, GA, USA; ^2^Department of Dermatology, Emory University, Atlanta, GA, USA; ^3^Grady Health System, Atlanta, GA, USA; ^4^Department of Pathology, Emory University, Atlanta, GA, USA; ^5^Veterans Affairs Medical Center, Atlanta, GA, USA

## Abstract

A 24-year-old female presented with granulomatous slack skin (GSS) cutaneous T-cell lymphoma. The patient underwent systemic chemotherapy. Owing to the development of several chemotherapy-related complications, therapy was discontinued. Subsequently, disease progression was noted clinically. Our patient’s disease progression was clearly demonstrated by 18F-fludeoxyglucose positron emission tomography (PET)/CT findings. PET/CT imaging findings of GSS have not yet previously been reported. In this report, we present PET/CT characteristics of a patient with GSS.

## Case report

**Figure 1:**
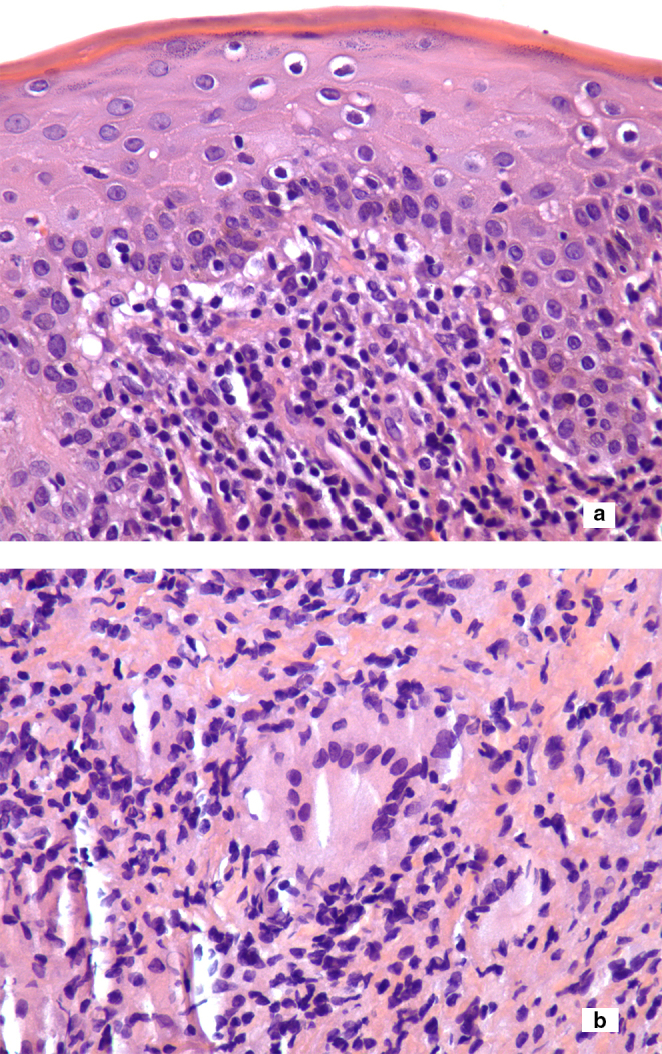
(a) Atypical lymphocytes infiltrate the epidermis (epidermotropism) (400×). (b) Non-necrotizing granulomatous inflammation in the dermis with multinucleated histiocytic giant cells, admixed with atypical lymphocytes (400×).

**Figure 2.**
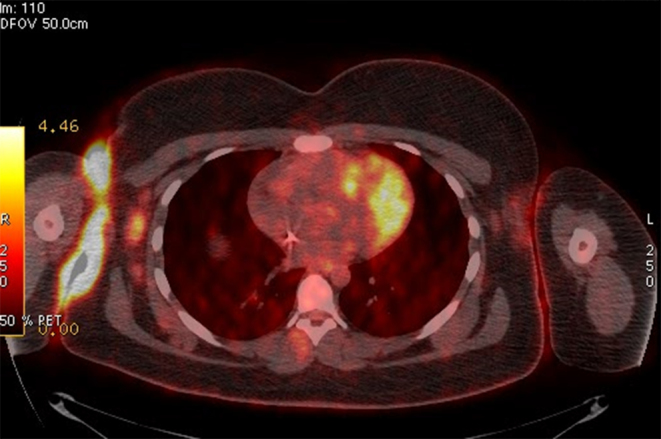
Axial fused positron emission tomography/CT image shows right axillary hypermetabolic adenopathy as well as marked skin thickening associated with hypermetabolic activity at the opposing skin folds of the right axillary region.

**Figure 3.**
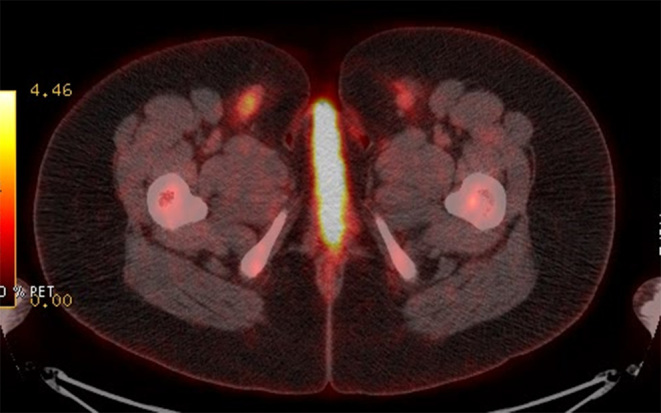
Axial fused positron emission tomography/CT image shows bilateral hypermetabolic inguinal lymph nodes and hypermetabolic activity of the vulvar region.

A 24-year-old black female presented with widespread skin changes on the upper and lower extremities, torso and genitalia. 2 years after the initial presentation, owing to worsening of her skin, she underwent partial vulvectomy. Lesional skin histopathology revealed the diagnosis of granulomatous slack skin cutaneous T-cell lymphoma (GSS CTCL) (Figure 1a,b). Over the course of 3 years, she received a variety of therapies. However, owing to therapy-related complications, she discontinued treatment. While off therapy, new skin lesions and lymphadenopathy developed. Our patient’s disease progression was clearly demonstrated by 18F-fludeoxyglucose (FDG) positron emission tomography (PET)/CT findings (Figures 2 and 3).

## Discussion

Primary CTCLs are a rare subgroup of non-Hodgkin lymphoma, with an annual age-adjusted incidence of approximately 6.4 per million persons.^1^ GSS is a very rare form of CTCL, approximately 50 cases of which have been reported to date.^2^ The role of PET/CT in assessing characteristics of CTCL has been recently studied.^3,4^ Although sites of cutaneous disease can be evaluated clinically, ^18^F-FDG PET/CT can help to direct biopsies to the most FDG-avid cutaneous disease site if large cell transformation is suspected.^4^ PET/CT also aids in the evaluation of extracutaneous involvement, specifically in the identification of possible nodal disease.^4^ Tsai et al^3^ also suggested that intensity of nodal FDG activity correlated with histologic lymph node grade.

GSS has been associated with the development of secondary lymphoid neoplasms, including Hodgkin lymphoma.^5^ Follow-up with PET/CT can be considered in selected cases.

PET/CT imaging findings of GSS have not yet previously been reported. In this report, we present PET/CT characteristics of a patient with GSS. Our case showed marked FDG activity of the skin lesions and identification of FDG-avid nodal disease, both of which supported the clinical evidence of disease progression.

## Learning points

1. PET/CT is useful to direct the site of biopsy in case of suspicion for large cell transformation of CTCLs.
2. PET/CT is useful to identify the sites of extracutaneous involvement of CTCLs.
3. As GSS has been associated with secondary lymphomas, PET/CT follow-up can be useful in selected cases.
